# Decoration of Silver Nanoparticles on WS_2_-WO_3_ Nanosheets: Implications for Surface-Enhanced Resonance Raman Spectroscopy Detection and Material Characteristics

**DOI:** 10.3390/molecules30030530

**Published:** 2025-01-24

**Authors:** Khaled Al Youssef, Adrien Chauvin, Jean-François Colomer, Carla Bittencourt

**Affiliations:** 1 Chimie des interactions Plasma-Surface (ChIPS), Materials Institute, University of Mons, 23 Place du Parc, 7000 Mons, Belgium; khaled.alyoussef@umons.ac.be (K.A.Y.); adrien.chauvin@eli-beams.eu (A.C.); 2 ELI Beamlines Facility, The Extreme Light Infrastructure ERIC, Za Radnicí 835, 25241 Dolní Břežany, Czech Republic; 3 Laboratory of Solid-State Physics (LPS), Namur Institute of Structured Matter (NISM), University of Namur, Rue de Bruxelles 61, 5000 Namur, Belgium; jean-francois.colomer@unamur.be

**Keywords:** tungsten disulfide WS_2_, tungsten trioxide WO_3_, vertically-aligned, surface-enhanced-Raman resonance scattering SERRS, Rhodamine B RhB, silver nanoparticles Ag(NPs), XPS, Raman, SEM

## Abstract

This study investigates the chemical and structural modifications of vertically aligned tungsten disulfide–tungsten trioxide (WS_2_-WO_3_) nanosheets decorated with silver nanoparticles (Ag(NPs)) under nitrogen plasma conditions. The synthesized vertically aligned WS_2_-WO_3_ nanosheets were functionalized through direct-current (DC) magnetron sputtering, forming silver-decorated samples. Structural changes, as well as the size and distribution of Ag(NPs), were characterized using scanning electron microscopy (SEM). Chemical state analysis was conducted via X-ray photoelectron spectroscopy (XPS), while Raman spectroscopy was employed to investigate vibrational modes. The findings confirmed the successful decoration of Ag(NPs) and identified unexpected compound transformations that were dependent on the duration of functionalization. The synthesized and functionalized samples were evaluated for their sensing capabilities towards Rhodamine B (RhB) through surface-enhanced resonance Raman scattering (SERRS). This study discusses the impact of substrate morphology and the shape and size of nanoparticles on the enhancement of SERRS mechanisms, achieving an enhancement factor (*EF*) of approximately 1.6 × 10^6^ and a limit of detection (LOD) of 10^−9^ M.

## 1. Introduction

Tungsten disulfide (WS_2_) is classified within the family of transition metal dichalcogenides (TMDs), characterized by the general chemical formula MX_2_, where M represents a transition metal such as molybdenum (Mo) or tungsten (W). X denotes a chalcogen element, including sulfur (S), selenium (Se), or tellurium (Te). TMDs are recognized for their unique properties, particularly their capacity to exist as thin-layered semi-conductors with tunable bandgaps that vary with layer thickness. The semi-conducting phase 2H-WS_2_ shows stability with a large direct band gap, which resulted in a large potential as a surface-enhanced Raman scattering (SERS) candidate [1].

Surface-enhanced Raman scattering (SERS) is an advanced analytical technique that enhances the vibrational fingerprints, usually detected by conventional Raman scattering, of adsorbed molecules by surface-metal-nanoparticle doping to identify chemical species [2,3,4]. WS_2_ showed a carrier mobility of approximately 140 cm^2^ V^−1^ s^−1^ at low temperatures and a high on/off current ratio of 10^6^ [5,6,7], revealing a pronounced photoluminescence (PL); [8] suggests a significant carrier density that plays a crucial role in the SERS effect through the chemical mechanism (CM). Additionally, tungsten trioxide (WO_3_) also exhibited considerable SERS efficiency. Nanostructured WO_3_ enriched with surface oxygen vacancies demonstrated an enhancement factor (*EF*) of up to 3.4 × 10^5^ and an LOD as low as 10^7^ M [9]. Moreover, the incorporation of oxygen into SERS-based MoS_2_ substrates resulted in enhancement factors of up to 10^5^ compared with oxygen-free MoS_2_ with an LOD of 10^−7^ M [10]. Thus, the development of SERS hetero-substrates composed of tungsten disulfide–tungsten trioxide (WS_2_-WO_3_) nanosheets seems appealing.

The functionalization of the SERS platform through doping with metal nanoparticles (MNPs) has proven efficiency in amplifying SERS signals due to the formation of hot spots—regions where the electromagnetic field is significantly intensified. Notably, silver nanoparticles (Ag(NPs)) are advantageous for interacting with analyte molecules, thereby enhancing the detection performance [11]. This enhancement is primarily attributed to localized surface plasmon resonance (LSPR) occurring at the valleys and sharp vertices of the substrate surface. In this context, vertically aligned nanosheet morphology leads to increasing active sites for hosting MNPs, allowed by the sharp edges and abundant vertices ultimately enhancing sensing performance [12,13,14].

D. Zhang et al. [15] investigated the SERS performance of WS_2_ monolayers decorated with silver nanoparticles (Ag(NPs)), noting a threefold increase in SERS signal following Ag incorporation. The enhancement was optimal for nanoparticle sizes ranging from 1 nm to 5 nm, while a decrease in enhancement occurred at 10 nm, attributed to a reduced boundary density compared to smaller Ag(NPs). Inspired by studies on protein coronas surrounding nanoparticles, Y. Song et al. [16] synthesized a monodispersed tungsten disulfide quantum dots modified silver nanosphere (Ag@WS_2_QD) through the hydrothermal method as a SERS substrate with a homogeneous distribution of hot spots. This group achieved an enhancement factor of up to four compared with colloidal Ag(NPs) when detecting thiram at a concentration of 10⁻^5^ M. In this study, vertically aligned WS_2_-WO_3_ nanosheets were synthesized through a chemical vapor deposition (CVD) process, followed by a two-step sulfurization. The deposition of Ag was conducted under nitrogen conditions, employing direct current magnetron sputtering as a physical vapor deposition (PVD) method. Functionalization was performed for varying durations of 5, 10, and 15 s, and the samples were prepared as SERS platforms for the detection of Rhodamine B (RhB). Characterization of the samples included the following: (i) surface morphology analysis via scanning electron microscopy (SEM), (ii) assessment of chemical state and surface elemental composition through X-ray spectroscopy, and (iii) vibrational fingerprints and modes by Raman.

## 2. Results

### 2.1. Scanning Electron Microscopy (SEM) for Morphology Investigation

**Figure 1:**
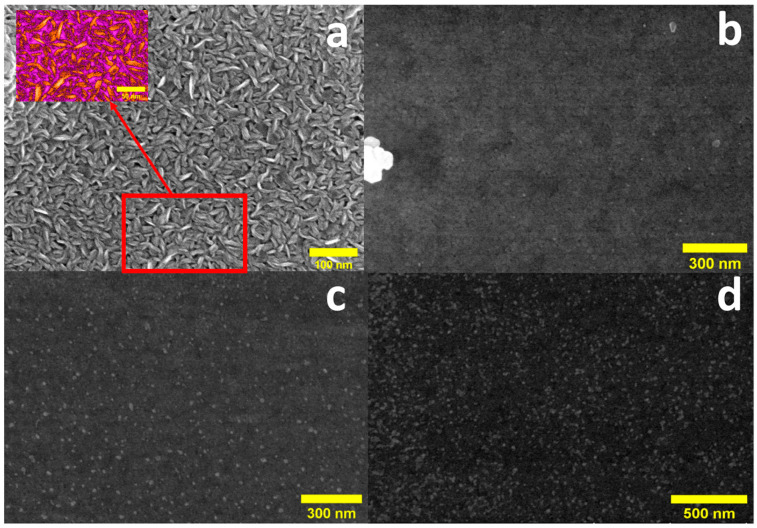
SEM images for (**a**) vertically aligned WS_2_-WO_3_, and after (**b**) 5 s, (**c**) 10 s, and (**d**) 15 s of Ag functionalization.

The morphology of WS_2_-WO_3_ is characterized by vertically oriented nanosheets, as illustrated in Figure 1a. The inset of Figure 1a highlights the distinct sharp top vertices of the vertically aligned nanosheets, indicating a wide range of sizes that results in their random arrangement across different levels. Following the functionalization, distinct glossy spots emerged on the surface after 5 s of silver deposition (sample N-VA-WS_2_(Ag_5_)), as depicted in Figure 1b. These spots indicate the presence of Ag(NPs). The Ag(NPs) are well dispersed across the top surface of the sample, albeit in low density (Figure S1), suggesting that the controllability of this functionalization can be modified to tune the surface-enhanced Raman scattering (SERS) efficiency. An increase in both the quantity and size of the Ag(NPs) was observed with prolonged functionalization time (Figure 1c,d). A uniform density distribution of particles is evident in the sample functionalized for 15 s N-VA-WS_2_(Ag15) (Figure 1d). The size distribution of the nanoparticles for different samples was fitted in a Gaussian distribution (Figure S2). The average particle size increases with increasing deposition time from 4 to 10 nm when increasing the functionalization from 5 to 15 s (Table 1).

The control of the size and density of Ag(NPs) decorating the surface of the active layer is essential for optimizing SERS enhancement [17]. During direct current (DC) magnetron sputtering, the adsorption of Ag atoms is significantly influenced by the morphological defects of the sample surface, particularly the presence of unsaturated edges and sharp vertices adjacent to dislocations. This phenomenon is believed to facilitate the proximity growth of nanoparticles, leading to their agglomeration into larger sizes [18,19], as observed in the N-VA-WS_2_(Ag_15s_). As the size of the surface nanoparticles increases, the inter-nanoparticle spacing decreases, which enhances the electromagnetic field generated by localized hotspots. This enhancement substantially amplifies the SERS signal [17]. Therefore, the optimization of the deposition parameters, including time, is critical for achieving desirable distributions and sizes of the nanoparticles.

### 2.2. X-Ray Photoelectron Spectroscopy (XPS) Analysis for Elemental and Chemical States

Comprehensive surface characterization was conducted on the vertically aligned WS_2_-WO_3_ samples and the functionalized counterparts using X-ray photoelectron spectroscopy to investigate the surface chemical states. All binding energies were calibrated with respect to the C1s peak centered at 284.8 eV [20] and normalized with respect to the W4f peak.

**Figure 2:**
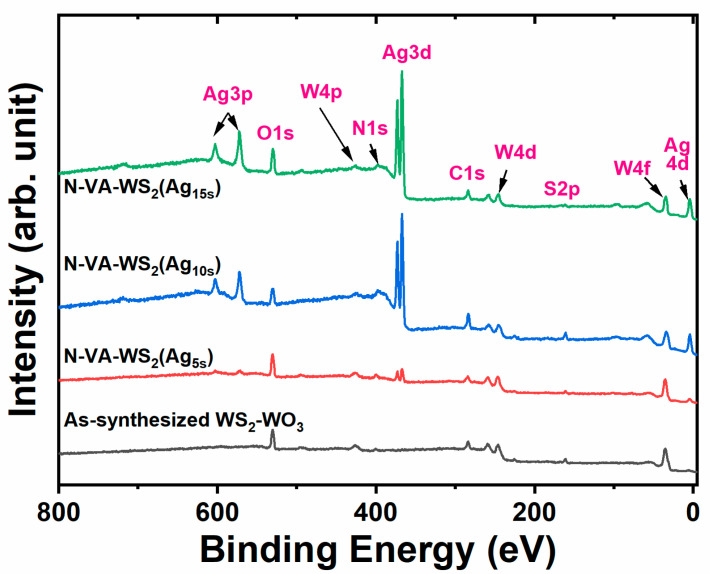
XPS survey spectra of the (black) as-synthesized WS_2_-WO_3_ and the Ag functionalized samples for (red) 5 s, (blue) 10 s, and (green) 15 s.

First, the survey spectra (Figure 2) revealed the presence of a doublet centered at ~368 eV and a singlet recorded at ~399.6 eV after 5 s of functionalization [21,22]. These peaks are attributed to the functionalization of the surface with Ag(NPs) and the grafting of nitrogen atoms. Notably, the intensity of these peaks exhibited a correlation with the duration of functionalization. The significant enhancement of the Ag peaks, despite the relatively brief functionalization time, can be ascribed to the vertically aligned morphology, which presents a high density of exposed edges that serve as active sites for silver nucleation.

Then, the X-ray photoelectron spectroscopy (XPS) survey spectra revealed a prominent O1s peak at ~531.5 eV. The intensity of this peak exhibited a slight decrease in the N-VA-WS_2_(Ag_10s_) sample, while an increase was observed in the N-VA-WS_2_(Ag_15s_) sample. A detailed core-level analysis via XPS was conducted for various elemental regions, specifically W4f, S2p, and N1s, which are centered at 32.5, 161.8, and 399.6, respectively [23].

**Figure 3:**
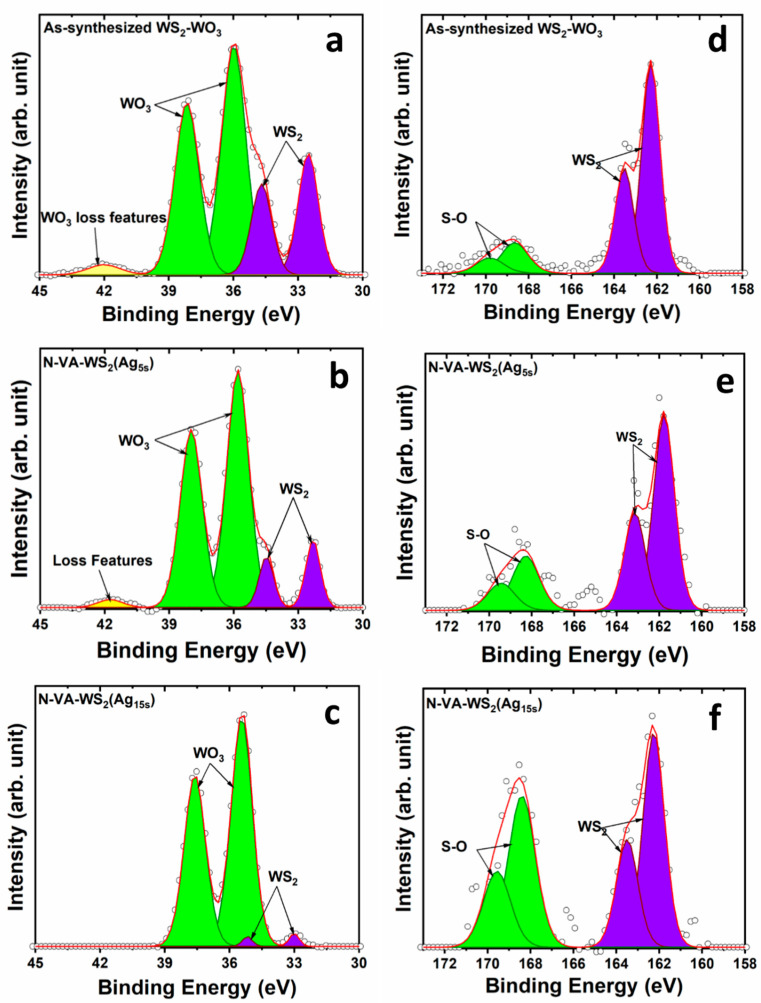
XPS core levels of (**a**–**c**) W4f and (**d**–**f**) S2p regions of the as-synthesized WS_2_-WO_3_, the functionalized N-VA-WS_2_(Ag_5s_), and N-VA-WS_2_(Ag_15s_), respectively. The open dots represent the data points, while the red line indicates the outcome of the fitting process.

The deconvolution of the W4f region was carried out using two doublets and one singlet (Figure 3a). The doublet with components at 32.5 eV and 34.5 eV is attributed to 4f_7/2_ and 4f_5/2_ orbitals of the semi-conducting phase 2H-WS_2_, respectively [23]. The oxide component is unambiguously distinguished by the doublet located at ~35.8 eV and 38.0 eV associated with 4f_7/2_ and 4f_5/2_ orbitals, respectively [15]. The peak located at ~42 eV is associated with the WO_3_ 5p_3/2_ loss feature. The WO_3_ state in the as-synthesized sample exhibited relatively intense peaks that increased with a longer time of functionalization, as seen for N-VA-WS_2_(Ag_5s_) and N-VA-WS_2_(Ag_15s_) in Figure 3b,c, respectively. The oxide component became predominant over the W4f region for 15 s of Ag deposition. The relative atomic concentration of WS_2_ and WO_3_ changed drastically when comparing the as-synthesized nanosheets with the ones subjected to 15 s of functionalization. In the as-synthesized sample, the $\frac{{\left(\%\right)\;WO}_{3}}{{\left(\%\right)\;WS}_{2}}$ ratio was ~2.3 and increased up to 38.5 in N-VA-WS_2_(Ag_15s_).

The S2p region was fitted using two doublets. The doublet with components located at ~162 eV and 163.5 eV for 2p_3/2_ and 2p_1/2_ orbitals, respectively, corresponds to the 2H-WS_2_, and the doublet representing S–O bonding was found at higher binding energy, i.e., 168.5 eV and 169.8 eV for 2p_3/2_ and 2p_1/2_ orbitals, respectively (Figure 3d). As seen in Figure 3e,f, the contribution of the component associated with oxygen bonding relatively increased after functionalization. After 15 s of Ag deposition, the S–O peaks became equally predominant in the S2p region.

**Figure 4:**
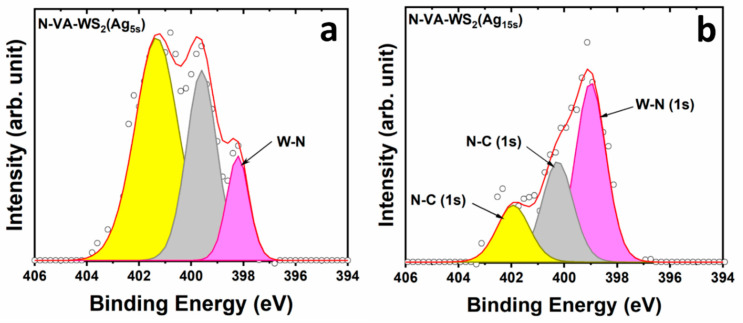
XPS core levels of N1s region of (**a**) the functionalized N-VA-WS_2_(Ag_5s_) and (**b**) the functionalized N-VA-WS_2_(Ag_15s_), respectively. The open dots represent the data points, while the red line indicates the outcome of the fitting process.

The N1s region, shown in Figure 4, was deconvoluted using three singlets centered at 398.2 eV, representing the chemical bonding of nitrogen with tungsten (W–N), 399.6 eV, and 401.3 eV for different carbon nitride bonding [24]. The relative intensity of the W–N component increased with increasing Ag deposition time, reflecting the increased amount of nitrogen grafted in the lattice surface (Figure 4a,b).

Regarding the Ag 3d region (Figure S3), the doublet peaks exhibited a Full Width at Half Maximum (FWHM) of 1.1 eV. Notably, sharp XPS peaks with an FWHM of less than 1 eV are indicative of bulk silver, whereas broader peaks suggest the presence of silver nanoparticles [25,26]. The observed decrease in the WS_2_ component can primarily be attributed to the incorporation of various species onto the substrate surface. The quantities of nitrogen and Ag incorporated onto the surface of the nanocomposite are summarized in Table 2. The observed abrupt increase in the intensity of the oxygen peak may be associated with the substrate morphology, which displayed a high density of exposed edges, rendering it highly reactive with oxygen. It is important to mention the absence of direct connections during the transfer of samples from the deposition and functionalization chambers to the X-ray photoelectron spectroscopy (XPS) chamber.

### 2.3. Valence Band Offset (VBO)

**Figure 5:**
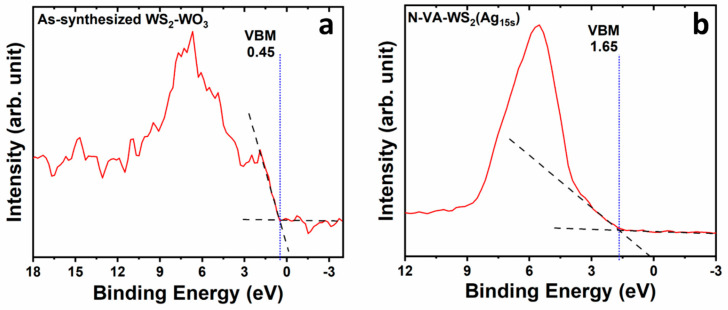
Valence bands of (**a**) the as-synthesized vertically aligned WS_2_-WO_3_, showing different orbital contribution and (**b**) the functionalized N-VA-WS_2_(Ag_15_) sample with quasi Ag VB. The solid red line is the outcome of the measured data.

The valence bands of the as-synthesized vertically aligned WS_2_-WO_3_ and the functionalized sample N-VA-WS_2_(Ag_15s_) are shown in Figure 5. The valence band maximum (VBM) is identified at the intersection of the baseline and the slope of the edge, located at 0.45 eV. WS_2_ contributes to the valence band through the W(5d) states, S(3p) states, and a prominent W(5d)–S(3p) coupling [27,28]. WO_3_ contributed to the valence band with O(2p) states. Notably, the shoulder observed at 1.5 eV is attributed to the substantial presence of oxygen, indicative of WO_3_ [29,30]. The valence band of the as-synthesized WS_2_-WO_3_ extends over approximately 10 eV (Figure 5a).

For the functionalized samples, the valence band became dominated by Ag(4d) states after 15 s of functionalization. This state exhibits a photoemission cross-section that is significantly greater than that of the W(5d) and S(3p) photoelectrons within the energy range employed during the X-ray photoelectron spectroscopy (XPS) measurements [31]. Consequently, this dominance limits the observation of contributions from the W(5d) and S(3p) states in the valence band. The valence band maximum (VBM) of N-VA-WS_2_(Ag_15s_) was determined to be 1.65 eV (Figure 5b). The valence band offset (VBO) was calculated relative to the W(4f)-to-valence band shift, yielding a value of 0.7 eV towards lower energy states, indicating a conductive n-type surface-enhanced Raman scattering (SERS) substrate. This shift may be associated with the increased presence of WO_3_ within the lattice. Tungsten oxide possesses a valence band maximum in close proximity to the conduction band minimum, which may facilitate the anticipated enhancement of the SERS signal by the chemical mechanism theory [32]. More information is in Figure S4.

### 2.4. Raman Spectroscopy for Structural Analysis

In the Raman characterization, low-energy modes were not considered, with a primary focus on two polarization-sensitive modes: the ${\;E}_{2g}^{1}$ and $A_{1g}$ modes. The $E_{2g}^{1}$ mode corresponds to the in-plane vibrational mode resulting from the opposing vibrations of sulfur (S) atoms relative to the tungsten (W) atom. In contrast, the $A_{1g}$ mode represents the out-of-plane vibrational mode [33,34], which arises from the vibrations of sulfur atoms along the *z*-axis (the vertical axis).

**Figure 6:**
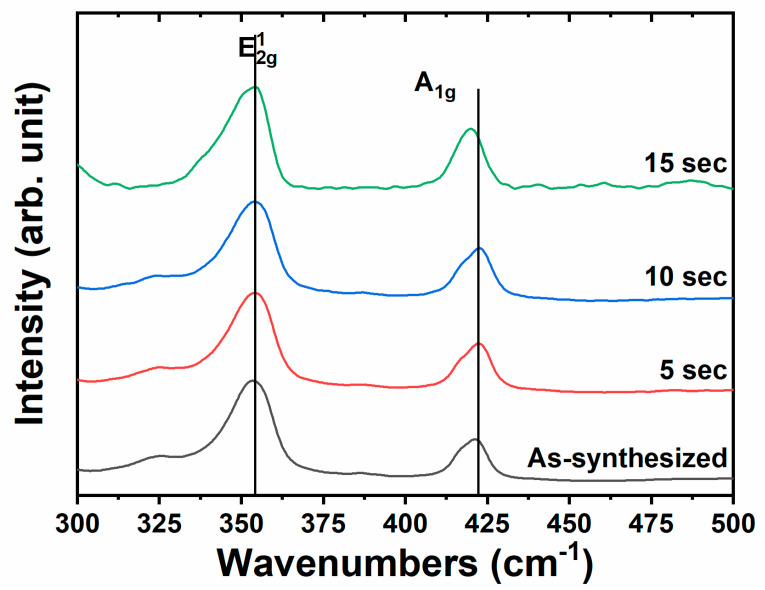
Raman spectra show different vibrational modes of the as-synthesized WS_2_-WO_3_ and the functionalized samples N-VA-WS_2_(Ag_5s_), N-VA-WS_2_(Ag_10s_), and N-VA-WS_2_(Ag_15s_).

Figure 6 shows spectra of the as-synthesized and the different functionalized samples. The $E_{2g}^{1}$ mode is observed at 353.5 cm^−1^, with no shift detected following the deposition process. The A_1_g mode is centered at 422 cm⁻^1^ for the as-synthesized sample, showing no changes in both functionalized samples, N-VA-WS_2_(Ag_5s_) and N-VA-WS_2_(Ag_10s_), while a slight redshift is noted for N-VA-WS_2_(Ag_15s_). This redshift may be attributed to the dielectric screening associated with the Stark effect [35]. Additionally, it could be related to surface etching during functionalization, which may lead to a reduction in the number of layers as the Raman spectrum is layer-dependent in this context. The frequency difference (∆ω) was reported to be 65.5 cm^−1^ for the monolayer WS_2_ and 70.5 cm^−1^ for the bulk counterpart [36,37]. The as-synthesized WS_2_-WO_3_ exhibited a ∆ω of 70.5 cm⁻^1^, indicative of a bulk-like sample that could be applied as a potential surface-enhanced Raman scattering (SERS) platform following optimal functionalization.

### 2.5. Surface-Enhanced Resonant Raman Scattering (SERRS) Measurement for the Detection of Rhodamine B

**Figure 7:**
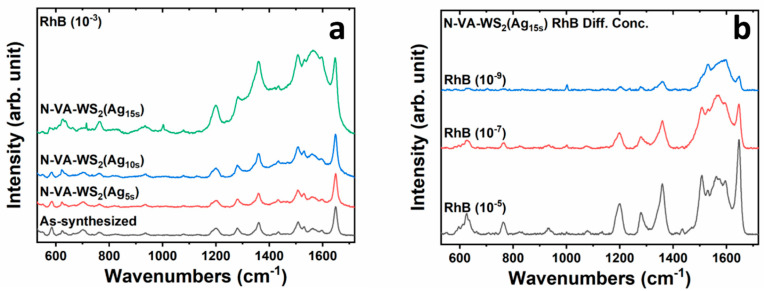
(**a**) SERRS spectra of RhB (10^−3^ M) detected on the as-synthesized WS_2_-WO_3_ nanosheets, the N-VA-WS_2_(Ag_5s_), the N-VA-WS_2_(Ag_10s_), and the N-VA-WS_2_(Ag_15s_). (**b**) SERRS spectra of RhB with different concentrations were recorded on N-VA-WS_2_(Ag_15s_).

The as-synthesized and functionalized substrates were evaluated as surface-enhanced resonant Raman scattering (SERRS) platforms for the detection of Rhodamine B (RhB) with a concentration of 10⁻^3^ M, as shown in Figure 7a. It is important to mention that the 532 nm excitation wavelength induces the Raman resonance of RhB since its absorption peak is located at 540 nm. This phenomenon significantly enhances SERS signals and is referred to as surface-enhanced resonance Raman scattering (SERRS) [38,39]. The Raman spectrum collected after the adsorption of RhB on the as-synthesized WS_2_-WO_3_ sample exhibits distinct, yet low-intensity band features characteristic of the detected analyte. These bands are identified in (Figure S4) and are in full agreement with previously reported results [40,41]. All spectra were normalized with respect to the A_1_g Raman mode of the underlying substrate material. This normalization procedure allows for a more accurate comparison of the relative intensities of the RhB-related bands across the different samples.

The SERRS spectra of Rhodamine B (RhB) collected on the functionalized samples, namely N-VA-WS_2_(Ag_5s_), N-VA-WS_2_(Ag_10s_), and N-VA-WS_2_(Ag_15s_), exhibit intense Raman bands. The most significant enhancement was observed for the sample functionalized for 15 s, N-VA-WS_2_(Ag_15s_), as shown in Figure 7a. This remarkable signal enhancement indicates the potential of the new hybrid nanocomposite materials as highly effective SERRS platforms. The substantial improvement in the SERRS response suggests that the specific functionalization conditions, particularly the 15 s treatment, have resulted in the creation of an optimal surface structure and morphology that can efficiently amplify the Raman scattering of the adsorbed RhB molecules. To further evaluate the sensitivity and limit of detection (LOD) of the N-VA-WS_2_(Ag_15s_) SERRS substrate, the detection of RhB at lower concentrations, including 10⁻^5^ M, 10⁻^7^ M, and 10⁻^9^ M, was tested. This assessment of the LOD will provide valuable insights into the practical applicability of this hybrid nanocomposite material for the ultrasensitive detection of trace-level analytes using SERRS technology.

The SERRS spectra of Rhodamine B (RhB) recorded on the N-VA-WS_2_(Ag_15s_) substrate at different analyte concentrations are shown in Figure 7b. The spectra exhibit all the distinct Raman bands of RhB reported in Table 3, even at the lowest concentration of 10⁻^9^ M. The enhancement factor (*EF*) of these SERRS measurements was evaluated using the following equation:

(1) $$EF=\frac{I_{SERRS}\times C_{i}\;}{I_{i}^{res}\times \;C_{SERRS}}$$

where $I_{SERRS}$ and $I_{i}^{res}$ are the relative SERRS signal intensities detected on the N-VA-WS_2_(Ag_15s_) nanocomposite and the as-synthesized vertically aligned WS_2_-WO_3_, referred to as the most intense band located at 1648 cm^−1^. Additionally, $C_{SERRS}$ and $C_{i}$ are the lowest RhB concentrations recorded on N-VA-WS_2_(Ag_15s_), and on the as-synthesized WS_2_-WO_3_, respectively [42,43]. The calculated *EF* values are presented in Table 3.

After 15 s of Ag functionalization, the WS_2_-WO_3_ substrate has reached an *EF* in the order of 10^6^ and a limit of detection (LOD) as low as 10⁻^9^ M. The significant enhancement is discussed in the following section.

## 3. Discussion

The signal enhancement observed in this study can be attributed to two primary mechanisms: the electromagnetic mechanism and the chemical mechanism. The electromagnetic mechanism involves the amplification of the electric field by several factors around the silver nanoparticles (Ag(NPs)) [44,45]. The vertically aligned topography provides sharp and dangling edges leading to the anisotropic surface (as seen in Figure 1a, where a large number of nanosheets with different heights are concentrated in a small area). This anisotropic surface structure results in a significant enhancement of the electric field through the generation of hotspots on the substrate surface after functionalization. This enhancement is supported by the work of Rycenga et al., which demonstrated that nanoparticles with sharp geometries and edges can lead to a greater enhancement of the electric field compared to spherical geometry [46]. Additionally, nanoparticles with nanotips and poly-edges have been shown to exhibit higher performance for optical applications than spherical nanoparticles [47]. Moreover, the optical performance is strongly dependent on the size of the nanoparticles [48]. Theoretical modeling revealed that the interaction between neighboring nanoparticles and between nanoparticles and the surface could significantly impact the energy potential, with larger nanoparticle sizes leading to a more pronounced increase in the energy potential between different alignments (face-to-edge) [49]. This is evidenced in Figure 1a, where the as-synthesized vertically aligned WS_2_-WO_3_ exhibits abundant and significant edges-to-face alignments. For smaller nanoparticle sizes, the potential energy was relatively weak, which may explain the SERRS signal enhancement of the N-VA-WS_2_(Ag_x_) samples with increasing nanoparticle size due to the functionalization time (Figure S2). Furthermore, the same study showed that the interaction between nanoparticles dominates the interaction between nanoparticles and the surface for larger nanoparticle sizes, supporting the current results obtained with the substrates. The anisotropic assembly of the substrate and the nanoparticles leads to efficient energetic hotspots that enhance the analyte SERRS signal [50,51,52,53]. Therefore, it is evident that the various factors, including the anisotropic bulk-like structure of the substrate and the anisotropic distribution of the nanoparticles, contribute to electromagnetic field enhancement, enabling the efficient detection of Rhodamine B (RhB).

The chemical mechanism can be attributed to the alteration of the valence band in the functionalized samples N-VA-WS_2_(Ag_x_). The impact of chemical enhancement can be crucial alongside electromagnetic enhancement. The contribution of the chemical enhancement is shown in Figure 5, where the valence band maximum (VBM) energy was shifted by 0.7 eV, leading to a reorganization of band states at the surface of the sample. This offset can be attributed to the increase in WO_3_ components in the samples. The WO_3_ VBM was recorded at 2.74 eV with a bandgap equal to 3 eV, which locates the Fermi level close to the conduction band [32,54,55]. This proximity of the band edges allows for efficient charge transfer between the substrate and the detected molecules, improving the SERRS signal by several folds. Additionally, the WS_2_-WO_3_ sample revealed a better electrochemical performance over WS_2_, which is attributed to the presence of oxide states that enhance the charge transfer process [56].

## 4. Materials and Methods

### 4.1. Chemicals

The Ag target used for nanoparticle deposition, with a diameter of 50.8 mm and a purity of 99.99%, was purchased from Advanced Engineering Materials, Changsha, China. The Tungsten Target was Ø 57 × 0.25 mm^2^, 99.95%, Mico to Nano, The Netherlands and the Sulfur Powder, precipitated, 99.5%, Alfa Aesar, Haverhill, MA, USA

The nitrogen reactive gas used for the sputtering was purchased from Air Liquide, Paris, France, and its purity is N_2_ ≥ 99,999%. Rhodamine B, with the empirical formula C_28_H_31_ClN_2_O_3_, used for the SERS experiment, was purchased from Sigma Aldrich, St. Louis, MO, USA, with a molecular weight of 479.01 g/mol.

The synthesized samples were labeled as follows: for the synthesized vertically aligned samples, WS_2_-WO_3_ nanosheets. For the functionalized samples, N-VA-WS_2_(Ag_x_), where N denotes nitrogen, VA is an abbreviation for vertically aligned, and x is the time used for silver deposition.

### 4.2. Synthesis of Vertically Aligned WS_2_-WO_3_ Nanosheets

WS_2_-WO_3_ nanosheets were synthesized by a double sulfurization chemical vapor deposition (CVD) atmospheric process reported in [57], using a Tungsten W film with a 50 nm thickness deposited onto a SiO_2_/Si substrate using DC-magnetron sputtering (Quorum Q15T/ES, East Sussex, UK). Tungsten W was sputtered in the presence of argon (99.9995% purity). Then, two steps of sulfurization were employed, where the deposited W film was placed along with sulfur S powder into the same reactor that was flushed with argon for one hour before sulfurization to remove/reduce oxygen. S was held in two zones (zone 1 at 40 °C (reached 400 °C after 30 min of the sulfurization process) and zone 2 at 850 °C). Subsequently, the obtained samples were cooled down with an argon flow for one hour.

### 4.3. Functionalization of the Vertically Aligned WS_2_-WO_3_ Nanosheets

The silver nanoparticles (Ag(NPs)) deposition under N-conditions was performed using a DC-pulsed magnetron sputtering RPG-50 from ONI, with a base pressure of 4 × 10^−6^ mbar along with the following parameters: mean power of P = 40 W, and a working pressure of P_w_ = 30 mTorr. Silver nanoparticles were deposited from a silver target on the cathode. The gases used were argon (Ar) as a carrier gas and nitrogen (N_2_) as a reactive gas. The gas flux was fixed to Φ = 20 sccm (Φ(Ar) = 2 sccm and Φ(N_2_) = 18 sccm). Samples were functionalized for different times: 5, 10, and 15 s.

### 4.4. SERS Procedures

An RhB stock aqueous solution with a concentration of 10^−3^ M was stored in darkness at 4 °C. Subsequently, different RhB solutions at different concentrations (10^−5^, 10^−7^, 10^−9^, and 10^−12^ M) were diluted from the stock. The as-synthesized and functionalized samples (0.5 cm × 0.5 cm) were tested through immersion into 5 mL of the aqueous solutions for 3 h. Afterward, all samples were thoroughly rinsed with deionized water, dried at a relatively low temperature of 20 °C for several minutes, and tested as SERS substrates.

### 4.5. Characterization of SERS Substrates

The morphology of both the as-synthesized and functionalized samples was examined utilizing scanning electron microscopy (SEM). This analysis employed the JEOL-JSM-7500F field-emission scanning electron microscope (JEOL, Tokyo, Japan), operated at an accelerating voltage of 2 kV, alongside the FESEM-SU8020-HITACHI (Hitachi, Tokyo, Japan), which is equipped with triple detectors and a Thermo Scientific NORAN System 7 X-ray detector (Waltham, MA, USA), functioning at 3 kV and 5 kV. The dimensions of the Ag(NPs) were determined by analyzing over 200 nanoparticles from each SEM image, employing ImageJ V1.53k software for quantitative assessment [58].

The chemical states and elemental compositions of the sample surfaces were investigated through X-ray photoelectron spectroscopy (XPS) conducted with the VERSAPROBE PHI 5000 instrument from Physical Electronics, Chanhassen, MN, USA. This analysis utilized an aluminum anode (Al Kα) X-ray radiation with an energy of 1486.6 eV. Measurements were performed at a takeoff angle of 45°, employing a hemispherical electron energy analyzer. The resulting data were analyzed using CasaXPS software V2.3.17.

Raman characterization was executed with a micro-Raman spectrometer (Senterra Bruker Optik GmbH, SENTERRA II, Berlin, Germany) at ambient temperature. The spectrometer was equipped with a color charge-coupled device (CCD) camera featuring a resolution of 1.3 megapixels. The optical system included a 50× objective lens with a working distance of 0.38 mm. The spectral range was established between 40 and 1540 cm⁻^1^, with a spectral resolution exceeding 3–5 cm⁻^1^ and an acquisition duration of 20 s. The excitation laser operated at a wavelength of 532 nm, delivering a power output of 2 mW. The excitation wavelength of 532 nm can induce Raman resonance in Rhodamine B (RhB), given that its absorption peak is located at 540 nm.

## 5. Conclusions

The morphology of the as-synthesized WS_2_-WO_3_ samples was characterized by vertically aligned nanosheets, which can be described as bulk-like. The functionalization of the as-synthesized material by silver under a nitrogen atmosphere was successfully performed, resulting in significant changes to the chemical and elemental composition at the surface. Ag(NPs) formed on the surface of the TMD nanosheets, exhibiting average sizes of 4.1 nm, 8.2 nm, and 10.6 nm for the N-VA-WS_2_(Ag_5s_), N-VA-WS_2_(Ag_10s_), and N-VA-WS_2_(Ag_15s_) samples, respectively. Moreover, the increase in W–N during functionalization was spotted. The new hybrid materials were evaluated as SERRS platforms for the detection of Rhodamine B (RhB). The vertically aligned functionalized substrates demonstrated significant enhancement in SERRS performance compared to the as-synthesized samples. The results were particularly notable for the N-VA-WS_2_(Ag_15s_) sample, which achieved an enhancement factor (*EF*) of approximately 1.6 × 10^6^ and a limit of detection (LOD) as low as 10^−9^ M. Several key factors contributed to the superior SERRS performance of the functionalized nanosheets. Primarily, the vertically aligned morphology and the incorporation of Ag(NPs) with various edge-surface alignments were critical. Additionally, the abundance of oxygen species on the surface likely improved the chemical enhancement process. Finally, the doping of the surface of the vertically aligned substrate by nitrogen simultaneously with Ag(NPs) opens the route towards more understanding of the effect of the substrate shape on their optical properties along with the surface chemical modifications. These findings highlight the important role of substrate morphology engineering in optimizing the performance of SERRS platforms for various applications, particularly in the sensitive detection and sensing of analytes.

## Figures and Tables

**Table 1:** Ag(NPs) size average of the functionalized VA-WS_2_ samples.

VA-WS_2_	Ag(NPs) Size Average (nm)
N-VA-WS_2_(Ag_5s_)	4.1 ± 0.6
N-VA-WS_2_(Ag_10s_)	8.2 ± 0.5
N-VA-WS_2_(Ag_15s_)	10.6 ± 0.5

**Table 2:** Atomic concentration percentages of Ag and N for N-VA-WS_2_ as functionalization time increases, as obtained by XPS data.

SERS Platform	Ag (%)	N (%)
N-VA-WS_2_(Ag_5s_)	4.1	9.8
N-VA-WS_2_(Ag_10s_)	21.0	7.0
N-VA-WS_2_(Ag_15s_)	26.1	7.0

**Table 3:** Relative intensities and concentrations were used to evaluate the *EF*.

Substrate	Relative Intensity (Arb. Unit)	Concentration (M)	*EF*
As-synthesized WS_2_-WO_3_	0.42	10^−3^	1.6 × 10^6^
N-VA-WS_2_(Ag_15s_)	0.66	10^−9^	

## Data Availability

The data presented in this study are available on request from the corresponding author.

## Supplementary Materials

The following supporting information can be downloaded at https://www.mdpi.com/article/10.3390/molecules30030530/s1. Figure S1: Silver nanoparticles (Ag(NPs)) showed by red arrows on the surface of the sample functionalized for 5 s, N-VA-WS_2_(Ag_5s_). Figure S2: Gaussian fitting of Ag(NPs) size for different functionalized samples as noted. Figure S3: XPS core level of Ag3d region of the functionalized N-VA-WS2(Ag_5s_) and the functionalized N-VA-WS2(Ag_15s_). Figure S4: Valence band structures of the functionalized samples N-VA-WS_2_(Ag5) and N-VAWS_2_(Ag10). The valence band maximum (VBM) of the as-synthesized WS_2_-WO_3_ is positioned at 0.45 eV. In the samples functionalized for 5 and 10 s, N-VA-WS_2_(Ag5) and N-VA-WS_2_(Ag10), the VBM is observed at 0.5 eV and 0.6 eV, respectively, indicating a slight shift towards lower energy states. In contrast, the sample N-VA-WS_2_(Ag15), which demonstrates significant SERS enhancement, exhibits a VBM of 0.7 eV. This observation suggests that tuning the valence band offset may play a pivotal role in influencing the SERS signal. Table S1: RhB vibrational modes detected by the as-synthesized WS_2_-WO_3_ and the different functionalized samples.

## Abbreviations

The following abbreviations are used in this manuscript:

WS_2_-WO_3_	tungsten disulfide–tungsten trioxide
Ag(NPs)	silver nanoparticles
DC	direct-current
SEM	scanning electron microscopy
XPS	X-ray photoelectron spectroscopy
RhB	Rhodamine B
SERS	surface-enhanced Raman scattering
EF	enhancement factor
LOD	limit of detection
TMDs	transition metal dichalcogenides
PL	photoluminescence
EM	electromagnetic mechanism
CM	chemical mechanism
MoS_2_	molybdenum disulfide
MNPs	metal nanoparticles
LSPR	localized surface plasmon resonance
QD	quantum dots
CVD	chemical vapor deposition
PVD	physical vapor deposition
VA	vertically aligned
N	nitrogen
eV	electron-volt
FWHM	Full Width at Half Maximum
VB	valence band
VBO	valence band offset
VBM	valence band maximum
∆ω	frequency difference
Ar	argon
sccm	Standard Cubic Centimeters per Minute
