# Supplementary material for: Decoration of Silver Nanoparticles on WS2-WO3 Nanosheets: Implications for Surface-Enhanced Resonance Raman Spectroscopy Detection and Material Characteristics
Source: Molecules. 2025 Jan 24;30(3):530. doi: 10.3390/molecules30030530 (PMC11820258; doi:10.3390/molecules30030530)
Supplement: Supplementary file 1 [file molecules-30-00530-s001.zip › molecules-3422179-supplementary.pdf]

Decoration of Silver Nanoparticles on WS2-WO3 Nanosheets: Implications for Surface-Enhanced Resonance Raman

Spectroscopy Detection and Material Characteristics

Khaled Al Youssef [1], Adrien Chauvin [1, 2], Jean-François Colomer[3] and Carla Bittencourt [1,*]

[1] Chimie des interactions Plasma-Surface (ChIPS), Materials Institute, University of Mons 23 Place du Parc, 7000 Mons, Belgium; e-mail : khaled.alyoussef@umons.ac.be

[2] ELI Beamlines Facility, The Extreme Light Infrastructure ERIC, Za Radnicí 835, 25241 Dolní Břežany, CzechRepublic

[3] Laboratory of Solid-State Physics (LPS), Namur Institute of Structured Matter (NISM), University of Namur, Rue de Bruxelles 61, 5000 Namur, Belgium

* Carla Bittencourt : e-mail : carla.bittencourt@umons.ac.be

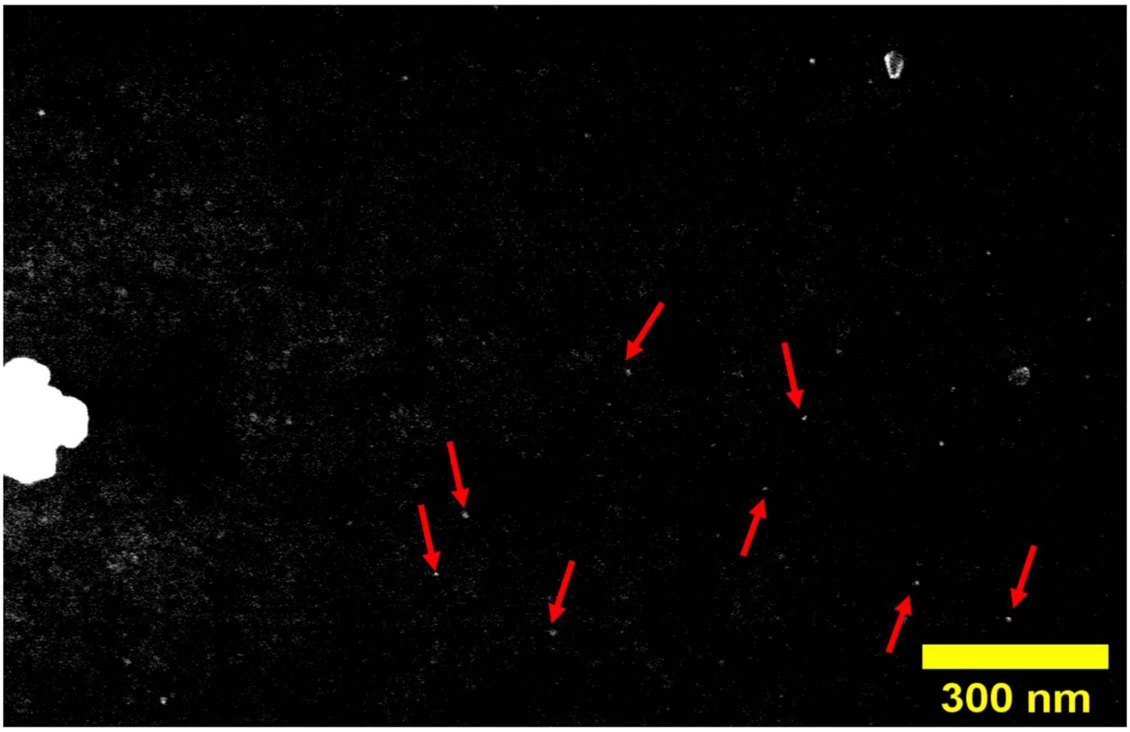

Figure S1. Silver nanoparticles (Ag(NPs)) showed by red arrows on the surface of the sample functionalized for 5 s, N-VA-WS_2(Ag_{5s}).

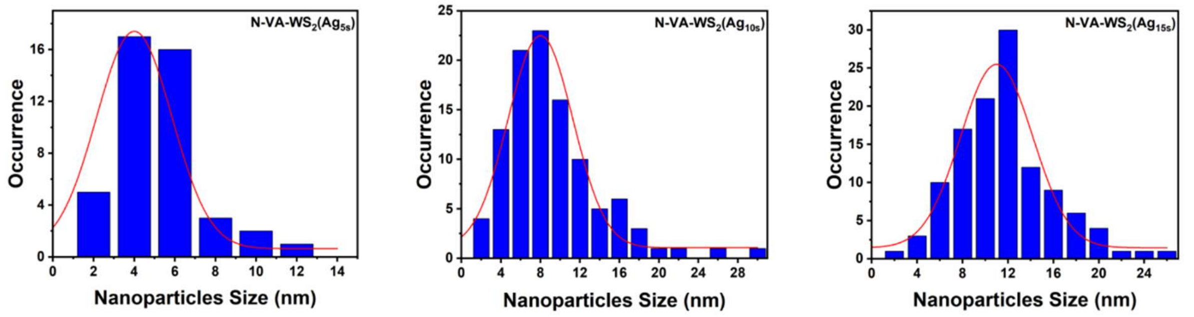

Figure S2. Gaussian fitting of Ag(NPs) size for different functionalized samples as noted.

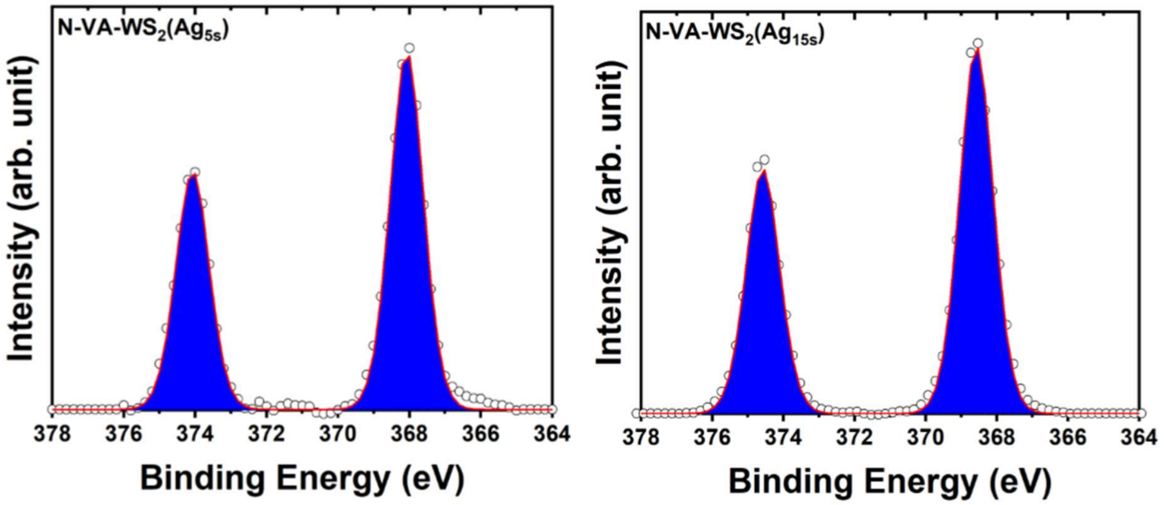

Figure S3. XPS core level of Ag3d region of the functionalized N-VA-WS_2(Ag_{5s}) and the functionalized N-VA-WS_2(Ag_{15s}).

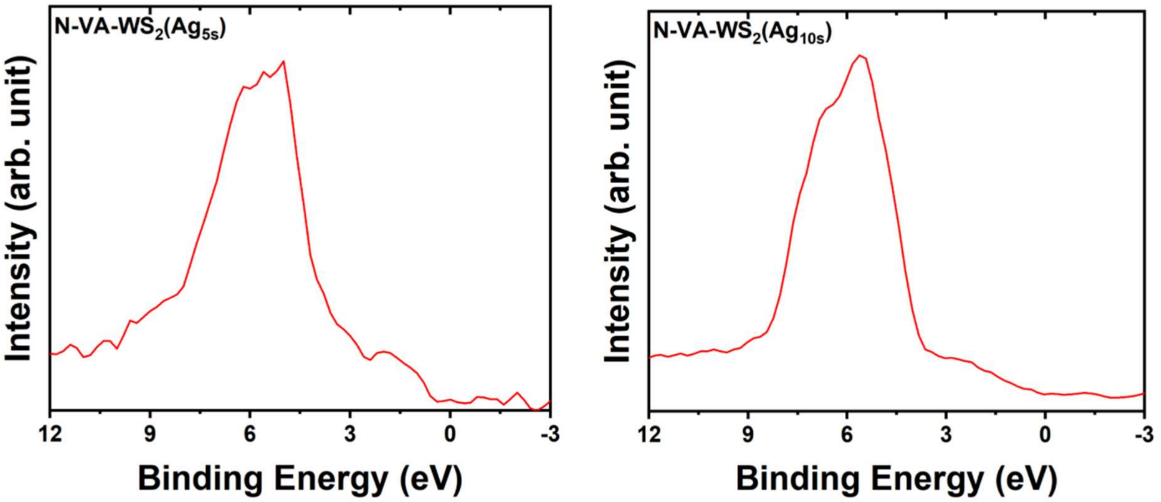

Figure S4. Valence band structures of the functionalized samples N-VA-WS_2(Ag5) and N-VA-WS_2(Ag10). The valence band maximum (VBM) of the as-synthesized WS_2-WO_3 is positioned at 0.45 eV. In the samples functionalized for 5 and 10 seconds, N-VA-WS_2(Ag5) and N-VA-WS_2(Ag10), the VBM is observed at 0.5 eV and 0.6 eV, respectively, indicating a slight shift towards lower energy states. In contrast, the sample N-VA-WS_2(Ag15), which demonstrates significant SERS enhancement, exhibits a VBM of 0.7 eV. This observation suggests that tuning the valence band offset may play a pivotal role in influencing the SERS signal.

Wave Number (cm^{-1})	Vibrational mode
625	C─C─C ring
760	C─H out-of-plane bending
943	C─H stretch
1195	C─H in-plane bending
1278	C─O─C stretching
1360, 1505, 1563 and 1648	C─C aromatic
1594	C═C stretching

Table S1. RhB vibrational modes detected by the as-synthesized WS_2-WO_3 and the different functionalized samples.
